# Single-atom Pt-I_3_ sites on all-inorganic Cs_2_SnI_6_ perovskite for efficient photocatalytic hydrogen production

**DOI:** 10.1038/s41467-021-24702-8

**Published:** 2021-07-20

**Authors:** Peng Zhou, Hui Chen, Yuguang Chao, Qinghua Zhang, Weiyu Zhang, Fan Lv, Lin Gu, Qiang Zhao, Ning Wang, Jinshu Wang, Shaojun Guo

**Affiliations:** 1 School of Materials Science and Engineering, Peking University, Beijing, P. R. China; 2 School of Materials and Energy, University of Electronic Science and Technology of China, Chengdu, P. R. China; 3 State Key Laboratory of Marine Resource Utilization in South China Sea, Hainan University, Haikou, P. R. China; 4 Institute of Physics, Chinese Academy of Sciences, Beijing, P. R. China; 5 The Beijing Innovation Center for Engineering Science and Advanced Technology, Peking University, Beijing, P. R. China

**Keywords:** Photocatalysis, Materials for energy and catalysis

## Abstract

Organic-inorganic lead halide perovskites are a new class of semiconductor materials with great potential in photocatalytic hydrogen production, however, their development is greatly plagued by their low photocatalytic activity, instability of organic component and lead toxicity in particular. Herein, we report an anti-dissolution environmentally friendly Cs_2_SnI_6_ perovskite anchored with a new class of atomically dispersed Pt-I_3_ species (PtSA/Cs_2_SnI_6_) for achieving the highly efficient photocatalytic hydrogen production in HI aqueous solution at room temperature. Particularly, we discover that Cs_2_SnI_6_ in PtSA/Cs_2_SnI_6_ has a greatly enhanced tolerance towards HI aqueous solution, which is very important for achieving excellent photocatalytic stability in perovskite-based HI splitting system. Remarkably, the PtSA/Cs_2_SnI_6_ catalyst shows a superb photocatalytic activity for hydrogen production with a record turnover frequency of 70.6 h^−1^ *per* Pt, about 176.5 times greater than that of Pt nanoparticles supported Cs_2_SnI_6_ perovskite, along with superior cycling durability. Charge-carrier dynamics studies in combination with theory calculations reveal that the dramatically boosted photocatalytic performance on PtSA/Cs_2_SnI_6_ originates from both unique coordination structure and electronic property of Pt-I_3_ sites, and strong metal-support interaction effect that can not only greatly promote the charge separation and transfer, but also substantially reduce the energy barrier for hydrogen production. This work opens a new way for stimulating more research on perovskite composite materials for efficient hydrogen production.

## Abstract (web summary)

Organic-inorganic lead halide perovskites are a new class of photocatalysts, however, instability and toxicity pose challenges. Here, the authors report a non-toxic all-inorganic Cs_2_SnI_6_ perovskite anchored with atomically dispersed Pt-I_3_ for efficient photocatalytic hydrogen production in hydrogen iodide solution.

## Introduction

Splitting hydroiodic acid (HI) has significant research value in the field of energy science and technology^1–5^. The traditional decomposition of HI at high temperature of 500 °C has been demonstrated to be an effective approach to produce hydrogen^2,5^, however, it is unsustainable, dangerous, and not cost-effective. Solar-driven splitting of HI, as a promising low-cost technique, has recently attracted more research interest because it can achieve the co-production of zero-emission hydrogen (H_2_) fuel and value-added chemicals (I_2_/I_3_^−^) only under light condition at room temperature (RT)^6–8^. The development of advanced photocatalysts is very necessary for achieving the high efficiency of photocatalytic HI splitting, however, unfortunately, the reported photocatalysts cannot stably work in HI solution with strong acid property, which poses a high demand on new material selection and photocatalyst design. Recently, the organic–inorganic lead halide perovskites (OLHPs), such as MAPbI_3_ (MA = CH_3_NH_3_), with the advantages of facile synthesis, low cost and superior optoelectronic characteristics^9–13^, have been proved to be a promising photocatalyst for hydrogen production. Nevertheless, the unstable organic component in those MAPbI_3_ photocatalytic materials easily suffers from the serious photo corrosion in HI solution^14–25^, which severely limit the applications of OLHPs in the photocatalytic HI splitting into hydrogen^19,21^. Besides, the lead toxicity of MAPbI_3_ also inhibits its practical application. In the seek for the lead-free perovskite materials, Sn-based perovskites have been demonstrated to have a narrower optical bandgap than that of the Pb-based perovskites^26–30^, indicating their larger light absorption range. Especially, all-inorganic Cs_2_SnI_6_ perovskite is more preferred material system in view of its good stability, superior conductivity, and appropriate energy band levels^31–35^.

Apart from above obstacles, the reported OLHPs-based photocatalysts still suffer from a very low photocatalytic activity caused by the serious photogenerated electron–hole recombination^15,17^, restricting their further development. Decorating cocatalyst onto semiconductors to form the hetero-structured photocatalysts has been regarded as one of the simplest and most effective strategies for inhibiting the charge recombination, and hence improving the photocatalytic performance in the H_2_ evolution reaction^36–40^. Considering the fact that halide perovskites possess a large number of defects caused by its inherent property of low temperature crystallization^41^, they might be ideal scaffolds to anchor metal atoms and stabilize single atoms for achieving the greatly enhanced photocatalysis. However, developing new procedures for achieving highly efficient photocatalysts of halide perovskites anchored by a new class of metal single atoms for the hydrogen production, to the best of our knowledge, is still a great challenge in the field of photocatalysis.

Herein, we demonstrate the first example on making a new class of single-atom Pt–I_3_ sites anchored on all-inorganic Cs_2_SnI_6_ perovskite (PtSA/Cs_2_SnI_6_) for efficient H_2_ evolution photocatalysis from HI splitting at RT via a facile and cost-effective strategy. Turnover frequency (TOF) of as-prepared PtSA/Cs_2_SnI_6_ catalyst exhibits 176.5-fold enhancements compared with Pt nanoparticle anchored on Cs_2_SnI_6_ (PtNP/Cs_2_SnI_6_) catalyst, outperforming all of reported Pt-loaded halide perovskites photocatalysts, along with excellent catalytic stability. Combining charge-carrier dynamics studies with theory calculations reveals that both unique coordination structure and electronic property of Pt–I_3_ sites and strong metal–support interaction (SMSI) effect are the main reasons in achieving the greatly enhanced photocatalytic performance in hydrogen production from HI aqueous solution.

## Results

### Energy band structure of Cs_2_SnI_6_ and its stability in aqueous HI solution system

**Fig. 1:**
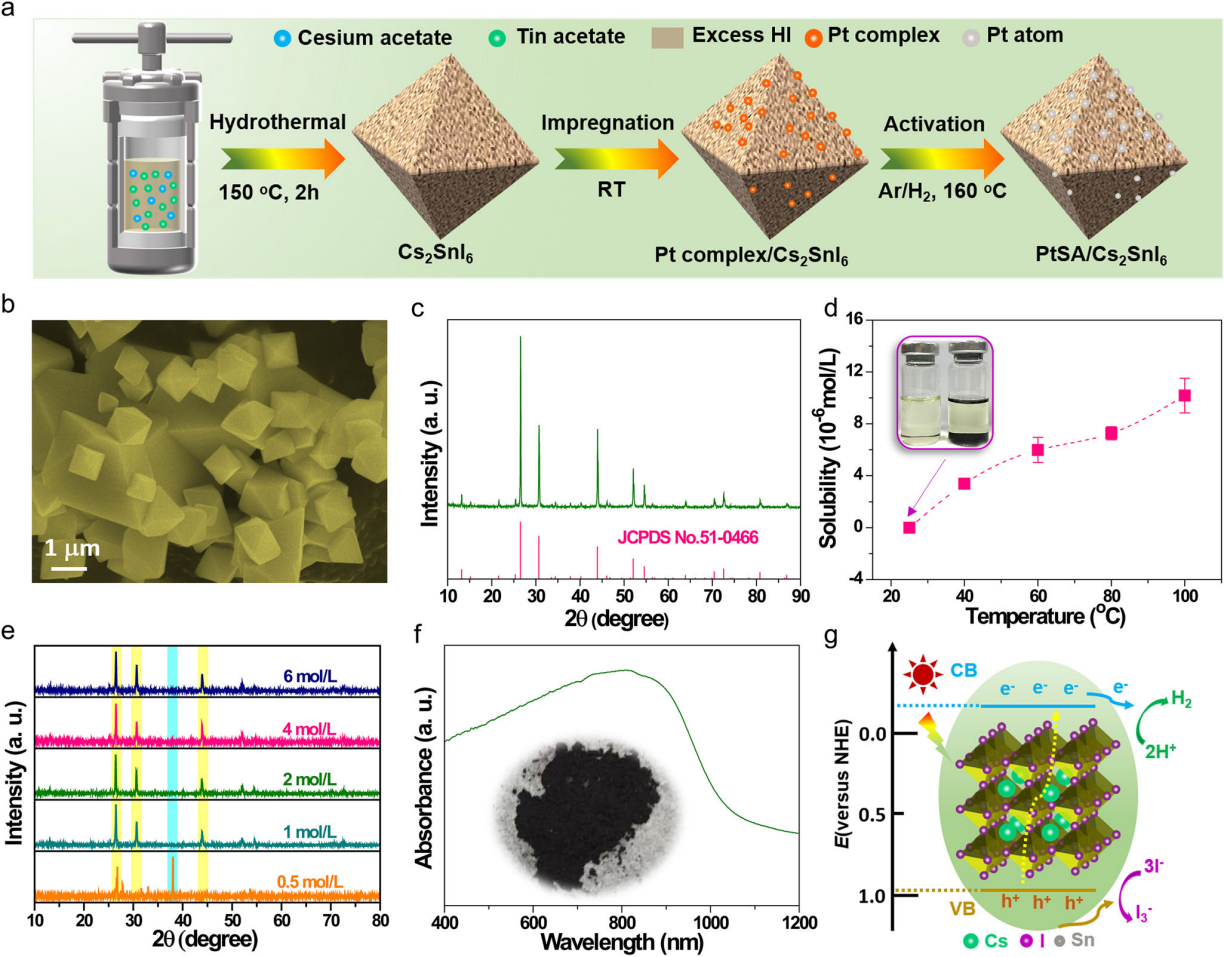
Energy band structure of Cs_2_SnI_6_ and its stability in aqueous HI solution system. **a** Schematic diagram of preparation process for the PtSA/Cs_2_SnI_6_ catalyst. **b** SEM image and **c** PXRD pattern of Cs_2_SnI_6_. **d** Solubility of Cs_2_SnI_6_ in aqueous HI solution at different temperature. The inset shows the photograph of the 6 M HI aqueous solution with and without Cs_2_SnI_6_ powder at 25 °C. **e** PXRD patterns of precipitates for Cs_2_SnI_6_ powder in aqueous HI solution with various concentrations. **f** UV–visible absorption spectrum of Cs_2_SnI_6_ powder. The inset is the photograph of the Cs_2_SnI_6_. **g** Schematic energy band diagram of the Cs_2_SnI_6_, charge generation and charge transfer process over the Cs_2_SnI_6_ under visible-light irradiation.

The synthetic procedure of PtSA/Cs_2_SnI_6_ is schematically illustrated in Fig. 1a. Briefly, the Cs_2_SnI_6_ was firstly synthesized by a one-pot hydrothermal treatment of cesium acetate and tin (II) acetate in presence of the excess hydriodic acid (HI) solution, followed by the impregnation of the platinum complex. Subsequently, the PtSA/Cs_2_SnI_6_ was obtained after activation at 160 °C for 1 h in H_2_/Ar atmosphere. Field-emission scanning electron microscopy image of the as-prepared Cs_2_SnI_6_ demonstrates that the Cs_2_SnI_6_ mainly adopts the octahedral morphology (Fig. 1b). Powder X-ray diffraction (PXRD) pattern (Fig. 1c) of the obtained Cs_2_SnI_6_ can be well indexed to the cubic Cs_2_SnI_6_ phase (JCPDS card NO. 51-0466), confirming the successful synthesis of Cs_2_SnI_6_. Furthermore, the as-synthesized Cs_2_SnI_6_ with a high yield of 93% exhibits the high-temperature stability with a decomposition temperature up to 350 °C in air atmosphere (Supplementary Fig. 1). Furthermore, Cs_2_SnI_6_ is insoluble in aqueous HI solution at RT, confirmed by the fact that the solubility of Cs_2_SnI_6_ was zero at 25 °C, and slightly increased to 10.0 × 10^−6^ mol L^−1^ as the temperature was improved to 100 °C, still far lower than that of reported MAPbI_3_ (0.645 mol L^−1^ at 20 °C) (Fig. 1d)^25^. Moreover, there is no change in the color of aqueous HI solution with or without Cs_2_SnI_6_ powder, which further verifies the insolubility for Cs_2_SnI_6_ (inset of Fig. 1d).

Furthermore, the stability of Cs_2_SnI_6_ in aqueous HI solution system was further investigated via PXRD (Fig. 1e). We find that the as-prepared Cs_2_SnI_6_ powder exhibits the excellent stability in aqueous HI solution system (decompose when HI concentration is <1.0 M), better than that of reported MAPbI_3_ (3.16 M)^25^. These results demonstrate that the Cs_2_SnI_6_-based aqueous HI splitting system is superior to the MAPbI_3_-based one, suggesting the great potential in photocatalytic HI splitting. The ultraviolet–visible (UV–vis) absorption spectrum and X-ray photoelectron spectroscopy (XPS) valence spectrum were employed to determine the energy band structure of as-prepared Cs_2_SnI_6_. As shown in Fig. 1f, the Cs_2_SnI_6_ powder has a broad optical absorption range, and its bandgap energy (*E*_*g*_) is 1.22 eV, by calculating from the absorbance data on the basis of the Kubelka–Munk equation, less than that of reported MAPbI_3_ (1.53 eV) (Supplementary Fig. 2)^25^. The result indicates that Cs_2_SnI_6_ possesses a larger light absorption range and higher conductivity than MAPbI_3_. The valence band (VB) position of the Cs_2_SnI_6_ is −5.46 eV with respect to the vacuum level (corresponding to 0.96 eV versus the normal hydrogen electrode (NHE)), as obtained from XPS VB spectroscopy (Supplementary Fig. 3). Accordingly, the corresponding conduction band (CB) position of Cs_2_SnI_6_, obtained by coupling the VB positions and *E*_*g*_, is calculated to be −4.24 eV with respect to the vacuum level (corresponding to −0.26 eV versus NHE). It should be noted that the calculated values of CB and VB are an approximation since the complicated electrolyte–perovskite interface effects were not considered herein. The suitable energy band levels of Cs_2_SnI_6_ (inset of Fig. 1g) provides new opportunity for straddling the redox potentials of HI to split HI into H_2_ and I_3_^−^.

### Structure characterization of PtSA/Cs_2_SnI_6_

**Fig. 2:**
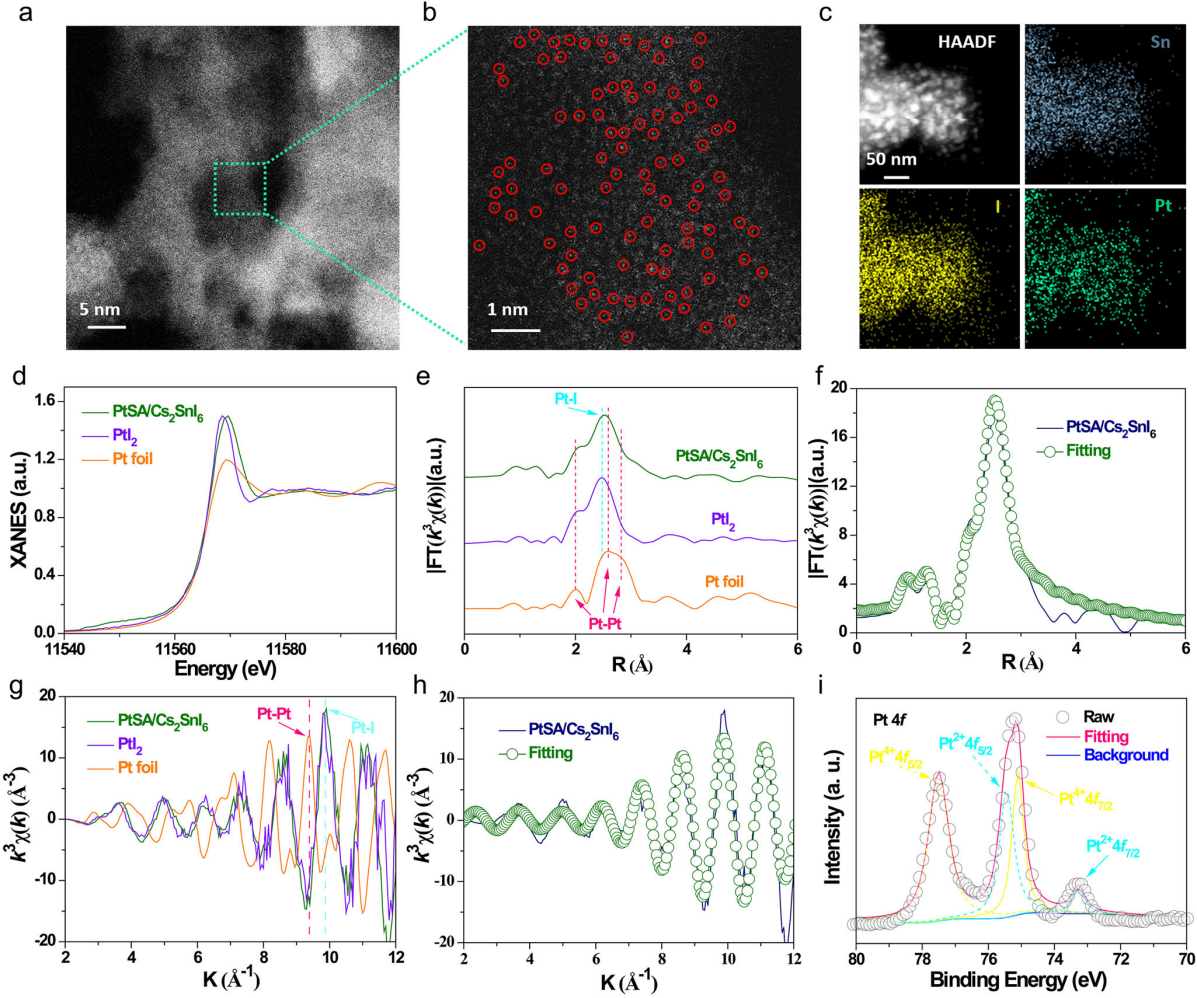
Structure characterization of PtSA/Cs_2_SnI_6_. **a** Low-magnification and **b** high-magnification HAADF-STEM images, and **c** the corresponding STEM-EDS elemental mapping of PtSA/Cs_2_SnI_6_. **d** Pt L_3_-edge XANES spectra and corresponding K^3^-weighted FT spectra at **e** R space and **g** k-space of PtSA/Cs_2_SnI_6_, PbI_2_ and Pt foil. XANES **f** R space and **h** k-space fitting curves of PtSA/Cs_2_SnI_6_. **i** High-resolution XPS Pt 4*f* spectrum of PtSA/Cs_2_SnI_6_.

The aberration-corrected high-angle annular dark field-scanning transmission electron microscopy (HAADF-STEM) was performed to confirm the distribution and configuration of Pt single-atom in Cs_2_SnI_6_. No Pt clusters or large nanoparticles were observed in the low-magnification HAADF-STEM image (Fig. 2a), further confirmed by PXRD analysis (Supplementary Fig. 4), in which there are no characteristic peaks of crystalline Pt species. The atomic-resolution HAADF-STEM image (Fig. 2b) clearly depicts the individual dispersion of Pt atoms on the surface of as-prepared Cs_2_SnI_6_. The corresponding energy dispersive X-ray spectroscopy (EDS) mapping images confirm the uniform dispersion of Sn, I, and Pt atoms in PtSA/Cs_2_SnI_6_ (Fig. 2c), indicating that Pt atoms are uniformly distributed over Cs_2_SnI_6_. The Pt content on PtSA/Cs_2_SnI_6_ is determined to be 0.12 wt% by inductively coupled plasma-atomic emission spectrometry (ICP-AES).

The oxidation state and coordination environment of the Pt species in PtSA/Cs_2_SnI_6_ were further investigated by X-ray absorption fine structure (XAFS) spectroscopy. Figure 2d depicts the normalized Pt L_3_-edge X-ray absorption near-edge structure (XANES) spectra of PtSA/Cs_2_SnI_6_, PtI_2_ and Pt foil. The white-line intensity of Pt L_3_-edge XANES of PtSA/Cs_2_SnI_6_ is higher than those of PtI_2_ and Pt foil, indicating that the oxidation state of Pt in PtSA/Cs_2_SnI_6_ is more than +2. The Fourier-transformed (FT) k^3^-weighted extended X-ray absorption fine structure (EXAFS) spectra (Fig. 2e) exhibits a prominent peak at ∼2.49 Å in PtSA/Cs_2_SnI_6_, similar to that of the Pt–I bond in PtI_2_, and shorter than that of the Pt–Pt bond in Pt foil, implying that the Pt atoms are anchored onto the surface of PtSA/Cs_2_SnI_6_ by the Pt–I bond. According to the EXAFS fitting results (Fig. 2f, Supplementary Figs. 5, 6, and Table 1), one Pt atom in PtSA/Cs_2_SnI_6_ is coordinated with approximately three I atoms (labeled as Pt–I_3_). To further confirm the Pt single atoms in the PtSA/Cs_2_SnI_6_, the wavelet transform (WT) EXAFS of the PtSA/Cs_2_SnI_6_ and the reference systems (PtI_2_ and Pt foil) was performed (Fig. 2g, h and Supplementary Figs. 7, 8). The WT-EXAFS maximum is observed at 9.38 Å^−1^ in Pt foil, assigned to the Pt–Pt bond. In contrast, the PtSA/Cs_2_SnI_6_ presents an intensity maximum value at 9.89 Å^−1^, similar to that of Pt–I bond in PtI_2_ (9.82 Å^−1^), further indicating the atomic dispersion of Pt coordinated with I atoms on the surface of PtSA/Cs_2_SnI_6_.

To further confirm the chemical state of Pt and chemical environment of Cs, Sn, and I in the catalysts, XPS measurements were conducted. As shown in Fig. 2i, the binding energy of Pt 4*f* in the high-resolution XPS spectrum of PtSA/Cs_2_SnI_6_ can be deconvoluted into two chemical states, assigned to Pt^2+^ at 73.1 and 75.6 eV, and Pt^4+^ at 75.1 and 77.5 eV, respectively, suggesting that Pt in the PtSA/Cs_2_SnI_6_ is Pt^δ+^ (2 < *δ* < 4), in agreement with the aforementioned Pt L_3_-edge XANES results. Noticeably, a positive shift in the I 3*d* XPS spectra (Supplementary Fig. 9a) is observed upon Pt loading, implying the strong interaction between the Pt and I atoms in PtSA/Cs_2_SnI_6_. It means that those I species can serve as the anchor sites, and coordinate with the isolated Pt atoms. Moreover, the binding energy of Sn 3*d* in PtSA/Cs_2_SnI_6_ has an obviously positive shift, whereas that of Cs 3*d* was unchanged relative to that in pure Cs_2_SnI_6_ (Supplementary Fig. 9b, c), implying that the electron transfer from Cs_2_SnI_6_ to PtSA depends on the Sn–I–Pt bonds rather than the Cs–I–Pt bonds.

### Superior photocatalytic activity and stability of PtSA/Cs_2_SnI_6_ catalyst

**Fig. 3:**
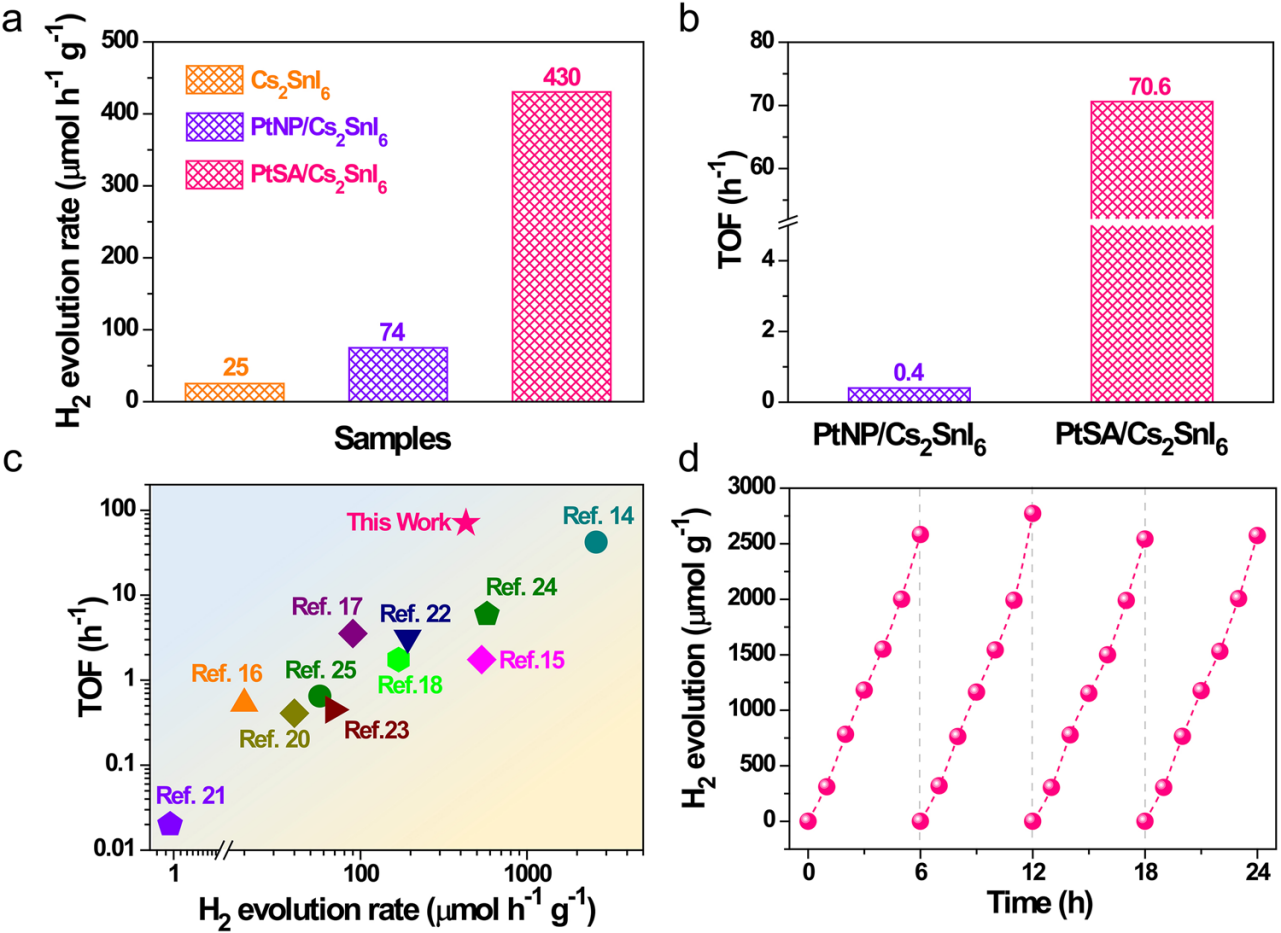
Superior photocatalytic activity and stability of PtSA/Cs_2_SnI_6_ catalyst. **a** The rate of photocatalytic H_2_ evolution over PtSA/Cs_2_SnI_6_, PtNP/Cs_2_SnI_6_, and Cs_2_SnI_6_ catalysts. **b** TOF of PtSA/Cs_2_SnI_6_ and PtNP/Cs_2_SnI_6_ catalysts. **c** TOF comparisons of PtSA/Cs_2_SnI_6_ catalyst and other reported Pt-loaded halide perovskite photocatalysts. **d** Cyclic stability of the PtSA/Cs_2_SnI_6_ catalyst.

The activities of photocatalytic H_2_ evolution over PtSA/Cs_2_SnI_6_, PtNP/Cs_2_SnI_6_, and Cs_2_SnI_6_ catalysts were evaluated in aqueous HI solution system under visible-light (*λ* ≥ 420 nm, 100 mW cm^−2^) irradiation by a 300 W Xe lamp and a homemade double-layered Pyrex vessel. The results show that pristine Cs_2_SnI_6_ owns a poor photocatalytic performance with a H_2_ production rate of 25 μmol h^−1^ g^−1^ (Fig. 3a and Supplementary Fig. 10). We find that the photocatalytic performance of Cs_2_SnI_6_ can be dramatically increased by anchoring Pt single atoms onto the surface of Cs_2_SnI_6_, reaching its maximum upon Pt loading up to 0.12 wt% (Fig. 3a and Supplementary Fig. 11). Further increasing or decreasing the loading content of Pt on Cs_2_SnI_6_ surface leads to the decreased photocatalytic activities. This is because the excessive Pt species can reduce the light absorption of Cs_2_SnI_6_ due to the shading effect whereas the insufficient Pt species cannot provide the rich H_2_-releasing active sites. Considering that the interfacial contact can affect the charge transfer from Cs_2_SnI_6_ to PtNP, we also prepared the PtNP on Cs_2_SnI_6_ (noted as PtNP_photo_/Cs_2_SnI_6_) by the direct photo-deposition method, and studied its photocatalytic activity. The result shows that the rate of H_2_ evolution (101 μmol g^−1^ h^−1^) over the optimal PtNP_photo_/Cs_2_SnI_6_ sample is only slightly better than that of PtNP/Cs_2_SnI_6_ (Supplementary Fig. 12), still much lower than that of PtSA/Cs_2_SnI_6_. The 0.12wt% PtSA/Cs_2_SnI_6_ shows the champion activity for the photocatalytic H_2_ production with a H_2_ production rate of 430 μmol h^−1^ g^−1^, 17.2 and 5.8 times higher than pure Cs_2_SnI_6_ and optimized 3.88wt% PtNP/Cs_2_SnI_6_ (Fig. 3a and Supplementary Figs. 13, 14), and also achieves a TOF of 70.6 h^−1^ per Pt, 176.5 times higher than that of PtNP/Cs_2_SnI_6_ catalyst (0.4 h^−1^) (Fig. 3b). Moreover, the TOF of PtSA/Cs_2_SnI_6_ catalyst is superior to all of the reported Pt-loaded halide perovskite photocatalysts (Fig. 3c and Supplementary Table 2).

Furthermore, the photocatalytic activity of PtSA/Cs_2_SnI_6_ can be stable without obvious decrease over four cycles and even at a successive 180-h tracking (Fig. 3d and Supplementary Fig. 15). After stability test, the Pt species are still atomically dispersed on the surface of Cs_2_SnI_6_, confirmed by the diverse results of PXRD, HAADF-STEM, and STEM-EDS elemental mappings (Supplementary Fig. 16–18). In addition, the XPS spectrum of Pt 4*f* in PtSA/Cs_2_SnI_6_ after photocatalytic test reveals that the valence of Pt species has no obvious change (Supplementary Fig. 19), revealing the excellent chemical stability of PtSA on Cs_2_SnI_6_.

### Charge-carrier dynamics

**Fig. 4:**
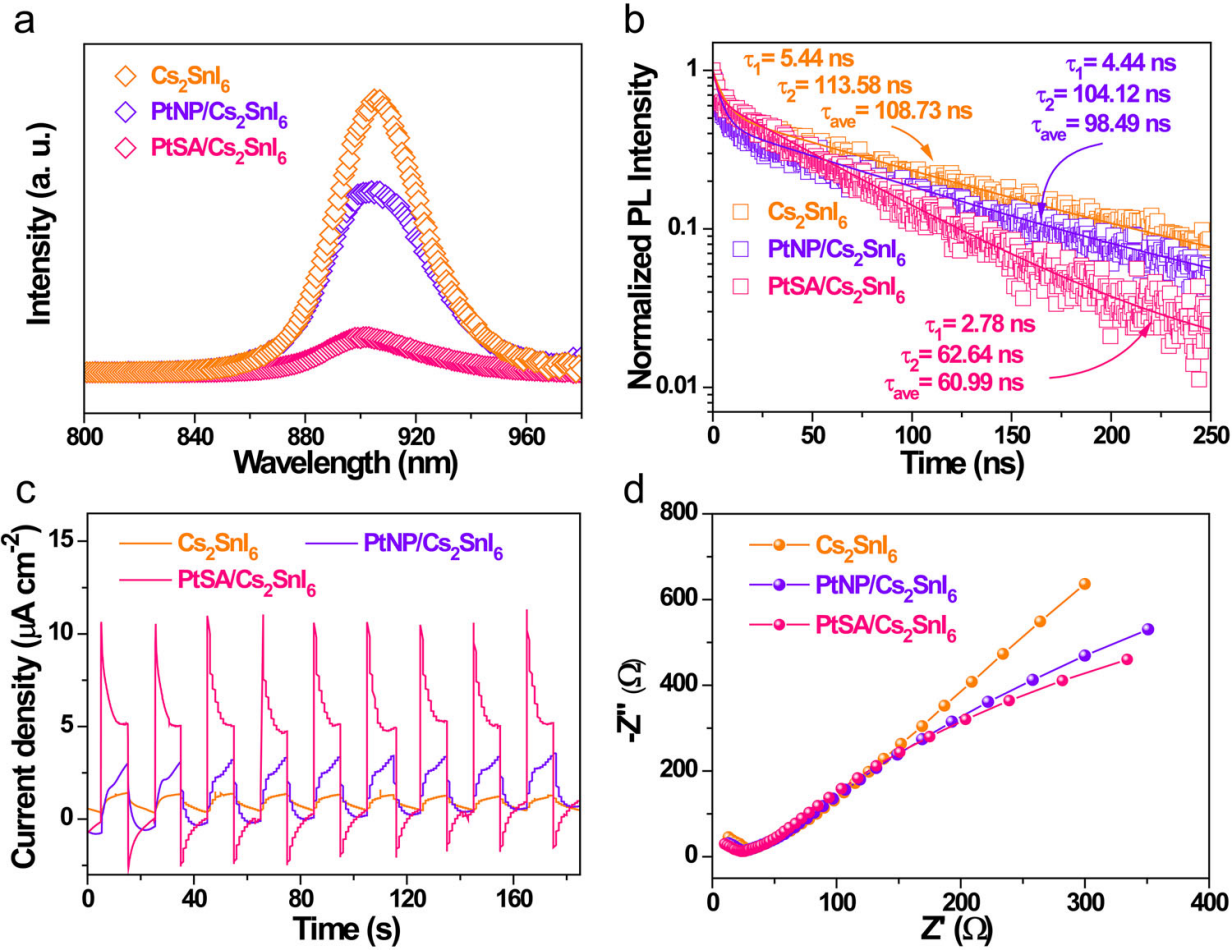
Charge-carrier dynamics. **a** Steady-state PL spectra, **b** time-resolved transient PL decay, **c** photocurrent responses spectra, and **d** electrochemical impedance spectroscopy of Cs_2_SnI_6_, PtNP/Cs_2_SnI_6_, and PtSA/Cs_2_SnI_6_ catalysts.

Photoluminescence (PL) technique was employed to evaluate the effect of Pt single atoms on the kinetics of charge carriers transfer and recombination over the Cs_2_SnI_6_. As depicted in Fig. 4a, the PL quenching of PtSA/Cs_2_SnI_6_ is more efficient than those of PtNP/Cs_2_SnI_6_ and Cs_2_SnI_6_, indicating the enhanced extraction and reduced recombination of charge carriers. To quantify the charge-carrier dynamic, TRPL spectroscopy was performed (Fig. 4b). The average decay lifetime of PtSA/Cs_2_SnI_6_ is ca. 61 ns (inset of Fig. 4b), smaller than those of PtNP/Cs_2_SnI_6_ (ca. 98 ns) and Cs_2_SnI_6_ (ca. 109 ns), further confirming that the atomically dispersed Pt species can enhance the separation of charge carriers. Besides, the photocurrent response spectra reveal that the PtSA/Cs_2_SnI_6_ exhibits a significantly enhanced photocurrent than those of PtNP/Cs_2_SnI_6_ and Cs_2_SnI_6_ (Fig. 4c), indicating its more efficient separation and transfer of photogenerated electron–hole pairs. Similarly, the linear-sweep voltammogram curves demonstrate that PtSA/Cs_2_SnI_6_ presents an overpotential of −0.38 V (versus RHE) at current density of 10 mA cm^−2^, much lower than those of PtNP/Cs_2_SnI_6_ and Cs_2_SnI_6_ (Supplementary Fig. 20), implying its higher efficiency during the surface catalytic reaction. Furthermore, the PtSA/Cs_2_SnI_6_ displays the smallest semicircle in Nyquist plots obtained from the electrochemical impedance spectroscopy (Fig. 4d), also suggesting that the atomically dispersed Pt–I_3_ configuration makes a great contribution to improving the efficiency of charge separation and transfer over Cs_2_SnI_6_.

### Theoretical calculations

**Fig. 5:**
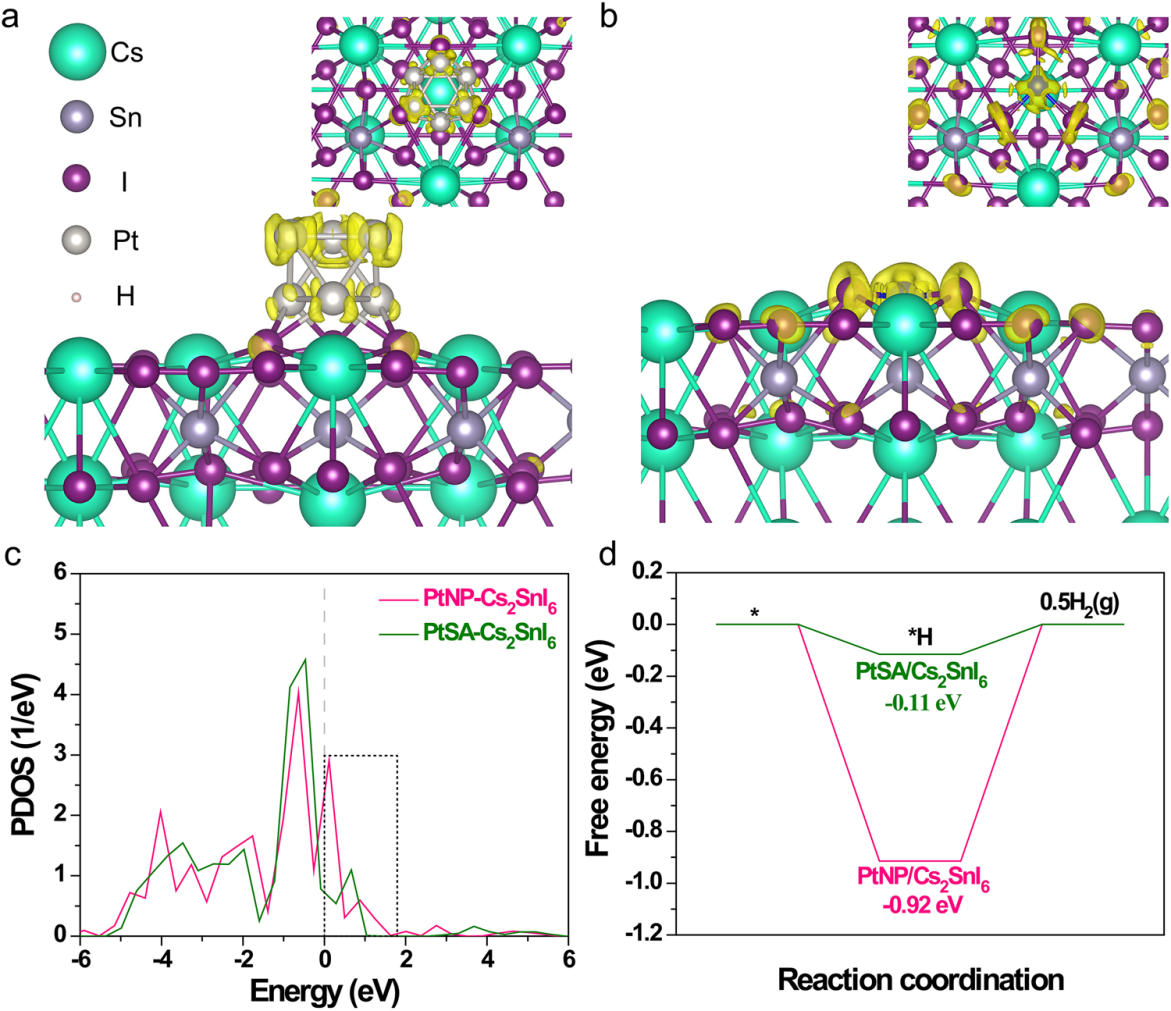
Charge density distribution and Gibbs energy calculations. The charge density difference maps between before and after photoexcitation: **a** PtNP/Cs_2_SnI_6_ and **b** PtSA/Cs_2_SnI_6_. The isosurface of charge density is 0.001e Å^−3^. The insets stand for the top view. The yellow region represents the additional electron distribution. An excess electron was added into the models, which was used to describe the photogenerated electron. **c** The PDOS (5*d* states) of PtNP/Cs_2_SnI_6_ and PtSA/Cs_2_SnI_6_. The dashed line stands for the Fermi level. **d** The calculated energy profile for hydrogen production on PtNP/Cs_2_SnI_6_ and PtSA/Cs_2_SnI_6_.

The photogenerated charge transfer on PtNP/Cs_2_SnI_6_ and PtSA/Cs_2_SnI_6_ was investigated by the DFT calculations. An extra electron was introduced to simulate the photogenerated electron in PtNP/Cs_2_SnI_6_ and PtSA/Cs_2_SnI_6_. The obtained charge density difference before and after photoexcitation reveals that the introduced photogenerated electrons tend to be distributed in the whole PtNP in PtNP-Cs_2_SnI_6_ (Fig. 5a), which undesirably decreases the electron density per Pt atom in PtNP. Instead, the electron in PtSA/Cs_2_SnI_6_ is only located between the PtSA and neighboring three I atoms (Fig. 5b), implying the high electron density on the Pt–I_3_ site. This is attributed to the SMSI effect^42^. Thus, the electron density per Pt atom in PtNP is further lower than that of PtSA, which is considered to lead to the lower HER activity of PtNP. To exclude the background charge effect, one donor hydrogen atom was introduced to calculate the charge density difference^43^. The obtained results also showed the localized distribution of electrons in the Pt–I_3_ region (Supplementary Fig. 21). This SMSI effect is beneficial to the photogenerated charge transfer between the photocatalyst and cocatalyst^42^, also confirmed by the calculated PDOS of PtNP and PtSA on Cs_2_SnI_6_ (Fig. 5c). The most 5*d* states of PtSA are below the Fermi level, indicating its electron-rich state. The integrated PDOS areas of uncaptured Pt 5*d* states above the Fermi level in PtNP and PtSA are calculated to be 1.19 and 0.71, respectively, which quantifies the different electron-saturation levels of PtNP and PtSA. This implies the relative electron-deficient property of PtNP. This means that the PtSA species owns a stronger ability for capturing electrons from the Cs_2_SnI_6_, leading to the higher hydrogen production activity of PtSA/Cs_2_SnI_6_. Thus the different electronic properties of PtNP and PtSA on Cs_2_SnI_6_ lead to their different catalytic dynamics in the H_2_ evolution process, in which the PtSA owns a remarkably lower energy barrier (0.11 eV) than PtNP (0.92 eV) (Fig. 5d). This well explains the observably higher photocatalytic H_2_ production activity of PtSA/Cs_2_SnI_6_.

## Discussion

In summary, we report a class of all-inorganic perovskite PtSA/Cs_2_SnI_6_ single-atom photocatalyst for achieving highly efficient photocatalytic hydrogen production in aqueous HI solution. The HAADF-STEM, XAFS spectroscopy, and XPS spectroscopy results confirm the atomically dispersed Pt single atoms with the well-defined Pt–I_3_ structure on Cs_2_SnI_6_. By combining charge-carrier dynamics studies and DFT calculations, we discover that the unique coordination structure and electronic property of Pt–I_3_ species can contribute to the SMSI effect, boosting the photogenerated electron transfer from Cs_2_SnI_6_ to Pt single atoms, and simultaneously reducing the Gibbs free energy and accelerating the kinetics for the hydrogen production. Benefiting from these structural advantages, an outstanding TOF of 70.6 h^−1^ per Pt was achieved over PtSA/Cs_2_SnI_6_ catalyst, 176.5-fold higher than that of PtNP/Cs_2_SnI_6_, setting a new TOF record reported for Pt-loaded halide perovskite photocatalysts, along with an excellent cycling stability. The achievements herein can significantly stimulate the exploitation of novel metal single atoms-perovskite hetero-structured photocatalysts systems and their further sustainable photocatalytic applications.

## Methods

### Synthesis of PtSA/Cs_2_SnI_6_

As-prepared Cs_2_SnI_6_ powder (0.10 g) was dispersed in 50 mL of chloroform containing different amount of Pt(acac)_2_. Then, the mixture was further ultrasonicated for 30 min and stirred for 12 h. Subsequently, the obtained precipitates were centrifugalized and washed for three times with isopropanol, and further dried in an oven 60 °C for 2 h. Finally, the PtSA/Cs_2_SnI_6_ was obtained by the treatment in a tube furnace at 160 °C for 1 h under a 5% H_2_/Ar atmosphere. The loading content of Pt on PtSA/Cs_2_SnI_6_ is determined by ICP-AES.

### XAFS characterization and data analysis

The Pt L_3_-edge XAFS spectra were performed at the 1W1B beamline of Beijing Synchrotron Radiation Facility (Beijing), operating at 2.5 GeV with a ring current of 250 mA. The X-ray beam was monochromatized by a Si (111) double crystal monochromator. The ATHENA module of the IFEFFIT software packages was used as the standard procedure to process the acquired EXAFS date. The k^3^-weighted *χ* (*k*) data of Pt K-edge in the k-space (2.0–12 Å^−1^) were FT to real (R) space by a handing windows (dk = 1.0 Å^−1^) to separate the EXAFS contribution from different coordination shells. The quantitative curve-fitting was performed by using the ARTEMIS module of IFEFFIT3 to obtain the elaborate structural parameters around Pt central atom in the as-synthesized catalysts. The functions of effective curved-wave backscattering amplitudes *F*(*k*) and phase shifts Φ(*k*) were calculated by the ab initio code FEFF8.0. Based on the fitting of reference samples of metal Pt bulk and PtI_2_ bulk, *S*_0_^2^ (amplitude reduction factor) was fixed to the best-fit value of 0.70. The interatomic distance (*R*) and the Debye–Waller factor (*σ*^2^) were allowed to change during the fitting analysis. The coordination of Pt–I was distinguished from Pt–Pt according to the bond length difference.

### Photocatalytic H_2_ evolution activity

The photocatalytic H_2_ evolution experiments in aqueous HI solution (containing 20 vol% H_3_PO_2_ as a stabilizer) were executed in a homemade double-layered Pyrex vessel. A 300 W Xe lamp with a visible-light illumination (*λ* ≥ 420 nm, 100 mW cm^−2^) was employed as a light source for the photocatalytic reaction. In a typical photocatalytic H_2_ evolution procedure, 10 mg of as-synthesized photocatalyst was introduced into 10 mL aqueous HI solution containing 20 vol% H_3_PO_2_ with the constant stirring rate, and then degassed with Ar through the reactor for 30 min to completely remove the dissolved air before irradiation. The reaction temperature was preserved at 25 °C by a circulation cooling water. The amount of evolved H_2_ was detected every hour in a 6 h test by gas chromatography (GC-7890B, Agilent, America, TCD, with MS-5 Å molecular sieve column) with Ar as the carrier gas. The recycling photocatalytic experiment for the stability test of as-prepared photocatalyst was carried out every 6 h as a cycle.

### Theoretical calculations

The photocatalytic property of PtSA/Cs_2_SnI_6_ was investigated by the Vienna Ab initio Simulation Package. The PAW pseudo-potentials were used to describe the interaction between valence electrons and the ionic core. The energy profile for hydrogen production was calculated by the revised Perdew–Burke–Ernzerh functional of the generalized gradient approximation. The reported standard hydrogen electrode (SHE) model was used in the calculations of Gibbs free energy changes (Δ*G*) in hydrogen adsorption^44^. In this model, the chemical potential (µ(H^+^) + µ(e^−^)) of a proton–electron pair was equal to half of the chemical potential (µ(H_2_)) of one gaseous hydrogen at *U* = 0 V versus SHE at pH = 0. The surface of Cs_2_SnI_6_ was simulated by its typical {111} facet, which was described by a 2 × 2 supercell with three Sn layers and six Cs–I layers (equal to three CsI–Sn–CsI layers). The PtSA was simulated by coordinating with three surface I atoms according to the above experimental results. The PtNP was described by a cluster containing 6 Pt atoms. The vacuum thickness was set to 16 Å. The larger supercells and higher vacuum thickness were tested, showing little effect on the calculation of hydrogen adsorption, as shown in Supplementary Table 3. A plane-wave basis with energy cutoff of 400 eV and an energy convergence threshold of 1.0 × 10^−5^ eV were used to perform the geometry optimization at the gamma point. After geometry optimization, the projected density of states of PtNP/Cs_2_SnI_6_ and PtSA/Cs_2_SnI_6_ models is calculated with the energy convergence of 1.0 × 10^−5^ eV and the Monkhorst–Pack k-point mesh of 2 × 1 × 1. To track the transfer of photogenerated electron between the Pt and photocatalyst, an extra electron with a compensating uniform background charge was used to simulate the photogenerated electron^43^. The energy convergence of 1.0 × 10^−5^ eV and the Monkhorst–Pack k-point mesh of 2 × 1 × 1 were adopted to calculate the difference of charge density plots between PtNP/PtSA and Cs_2_SnI_6_. Besides, to exclude the background charge effect caused by the extra electron, the charge difference density maps between PtNP/PtSA and Cs_2_SnI_6_ was also calculated by inserting a donor hydrogen atom into the Cs_2_SnI_6_ bulk, which can provide one proton and one electron^43^.

## Supplementary information

Supplementary Information

Peer Review File

## Data Availability

All experimental data within the article and its Supplementary Information are available from the corresponding author upon reasonable request.
