# Supplementary material for: Single-atom Pt-I3 sites on all-inorganic Cs2SnI6 perovskite for efficient photocatalytic hydrogen production
Source: Nat Commun. 2021 Jul 20;12:4412. doi: 10.1038/s41467-021-24702-8 (PMC8292376; doi:10.1038/s41467-021-24702-8)
Supplement: Supplementary file 1 — Supplementary Information [file 41467_2021_24702_MOESM1_ESM.pdf]

Supporting Information

Single-Atom Pt-I_3 Sites on All-Inorganic Cs_2SnI_6 Perovskite for Efficient Photocatalytic Hydrogen Production

Peng Zhou[1,†], Hui Chen[1,2,3†], Yuguang Chao[1,†], Qinghua Zhang[4], Weiyu Zhang[1], Fan Lv[1], Lin Gu[4], Qiang Zhao[2], Ning Wang[2,3], Jinshu Wang[2], Shaojun Guo [1,5*]

[1]School of Materials Science and Engineering, Peking University, Beijing 100871, P. R. China.

[2]School of Materials and Energy, University of Electronic Science and Technology of China, Chengdu 610054, P. R. China.

[3]State Key Laboratory of Marine Resource Utilization in South China Sea, Hainan University, Haikou 570228, P. R. China.

[4]Institute of Physics, Chinese Academy of Sciences, Beijing 100190, P. R. China.

[5]The Beijing Innovation Center for Engineering Science and Advanced Technology, Peking University, Beijing 100871, P. R. China.

[†]These authors contributed equally to this work

*Corresponding to: guosj@pku.edu.cn (S. G.)

Methods

Chemicals and materials. All reagents were used without further purification. Cesium acetate (99.99%), tin(II) acetate (99%), platinum(II) acetylacetonate ($Pt(acac)_2$, 99.98%), hydriodic acid solution (HI, 57 wt% in water), hypophosphorous acid solution (H_3PO_2, 50 wt% in water), isopropanol (99.9%), dichlormethane (99.8%) and chloroform (99.8%) were purchased from Sigma Aldrich. Tetrabutylammonium hexafluorophosphate ($TBAPF_6$, 98%), chloroplatinic acid hexahydrate ($H_2PtCl_6{\cdot}6H_2O$, ≥ 37.5% Pt basis) and poly (N-vinyl-2-pyrrolidone) (PVP, MW = 30000) were received from Macklin.

Synthesis of Cs_2SnI_6. The Cs_2SnI_6 powder was fabricated *via* a modified hydrothermal method[1]. Typically, cesium acetate (1.536 g) and tin(II) acetate (1.894 g) were dissolved in 20 mL of hydriodic acid solution, and then mixed in the solution with magnetic stirring for 30 min. After that, the mixed solution was transferred into a 50 mL Teflon-lined stainless steel autoclave and kept in an oven at 150 °C for 2 h. Next, the products were collected by centrifugation and washed for several times with isopropanol. Finally, the products were dried in an oven 60 °C for 6 h.

Synthesis of PtNP/Cs_2SnI_6. Pt nanoparticles (PtNP) were synthesized according to the previous reports[2]. Cs_2SnI_6 powder (0.10 g) and different amount of as-prepared PtNP were dispersed in 50 mL of isopropanol. The mixture was further ultrasonicated for 30 min and stirred for 6 h. The products were precipitated by centrifugation and dried in an oven 60 °C for 6 h.

Synthesis of $PtNP_{photo}/Cs_2SnI_6$. Typically, 0.1 g of Cs_2SnI_6 powder and calculated amount of H_2PtCl_6 solution (10 mg/mL) were dispersed in 50 mL of HI solution (containing 20 vol% H_3PO_2). Then, the mixture was exposed to a 300 W Xe-lamp with a 420 nm cutoff filter for 3 h at room temperature. The products were precipitated by centrifugation and dried in an oven 60 °C for 6 h.

Catalyst characterizations. FESEM and TEM images were performed using Zeiss LEO-1530, JEOL-JEM 2010 electron microscope, respectively. HAADF-STEM was conducted on JEMARM200F transmission electron microscope with an accelerating voltage of 300 kV and equipped with double spherical aberration (Cs) correctors. The EDS elemental mapping was recorded on STEM. PXRD

analysis was measured using Rigaku D/MAX-2500H X-ray powder diffractometer with Cu Kα radiation (40 KV, 100 mA). UV-Vis absorption spectra were performed by PerkinElmer UV-Lambda 950 instrument. Thermal stability of as-synthesized Cs_2SnI_6 powder was analyzed by thermogravimetric analysis (TGA) on a differential thermal analysis instrument (Q1000DSC +LNCS+FACS Q600SDT) under an air atmosphere. The steady PL and time-resolved PL decay measurements were performed by Edinburgh Instruments LTD (FLSP920) with an excitation wavelength at 535 nm at room temperature. Photoelectrochemical measurements were recorded by CHI electrochemical workstation (CHI66e, CH Instrument) in a three-electrode electrochemical cell configuration with as-synthesized photocatalyst as the working electrode, a Pt foil as the counter electrode and an Ag/AgCl electrode (with saturated KCl aqueous solution) as the reference electrode. The photocurrent responses were measured by utilizing 300W Xe lamp with an ultraviolet cut-off filter ($\lambda \geqslant 420$ nm) as the light source at 0 V *vs.* Ag/AgCl reference and 0.1 M $TBAPF_6$ dichlormethane solution as electrolyte. The LSV curves were measured in the HI and H_3PO_2 mixed solution (57 wt% HI 16 mL + 50 wt% H_3PO_2 4 mL) at a scan rate of 50 mV s^{-1}. EIS were conducted at open potential with frequency from 0.01 to 100,000 Hz. XPS measurements were measured on an Axi-ultra spectrometer with Al Kα X-ray source and low-energy electron flooding for charge compensation. The content of Pt was quantified on PerkinElmer Optima 7300DV ICP-AES.

Solubility and stability measurement of Cs_2SnI_6 in aqueous HI solution system. 100 mg of as-prepared Cs_2SnI_6 was added into 0.5 mL of aqueous HI solution system, and then ultrasonicated for 30 min at different temperatures. Next, the supernatant liquid was used to detect the tin ion concentration using inductively coupled plasma-atomic emission spectrometry (ICP-AES). 100 mg of as-prepared Cs_2SnI_6 was added into 0.5 mL of aqueous HI solution with various concentrations (0.5~6.0 mol L^{-1}) and then ultrasonicated for 10 min. Subsequently, the obtained precipitates were filtered and evaluated by PXRD.

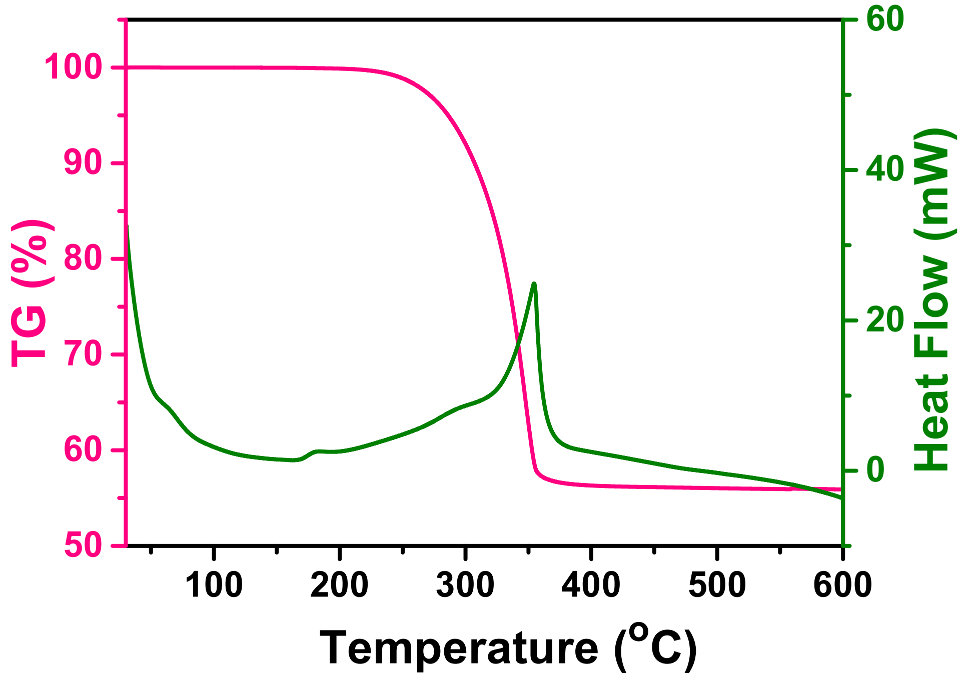

Supplementary Fig. 1 The thermogravimetry and differential scanning calorimetry of as-prepared Cs_2SnI_6 powder.

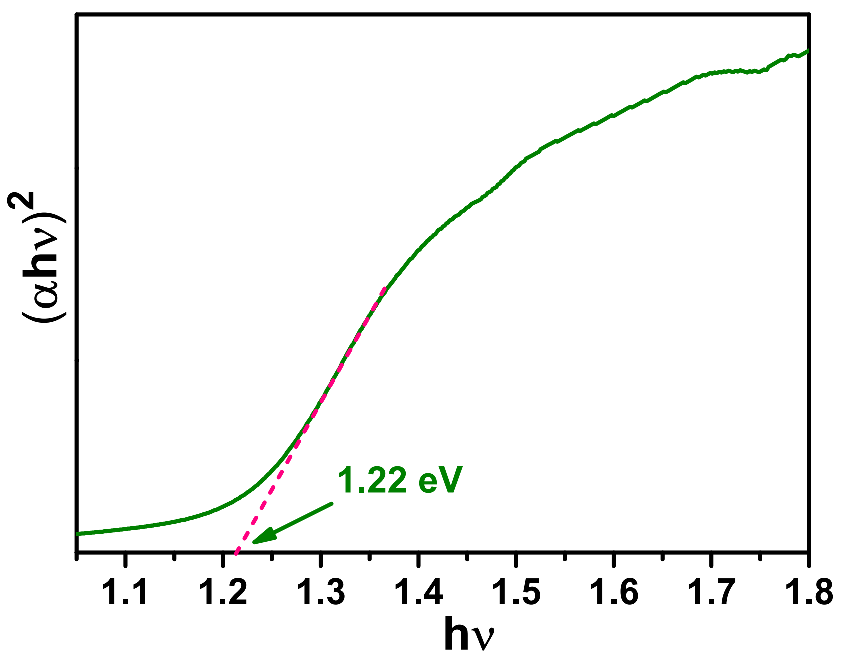

Supplementary Fig. 2 The $(\alpha h\nu)^2$-hν plot of as-prepared Cs_2SnI_6 powder obtained from absorption spectrum on the basis of the Kubelka-Munk equation.

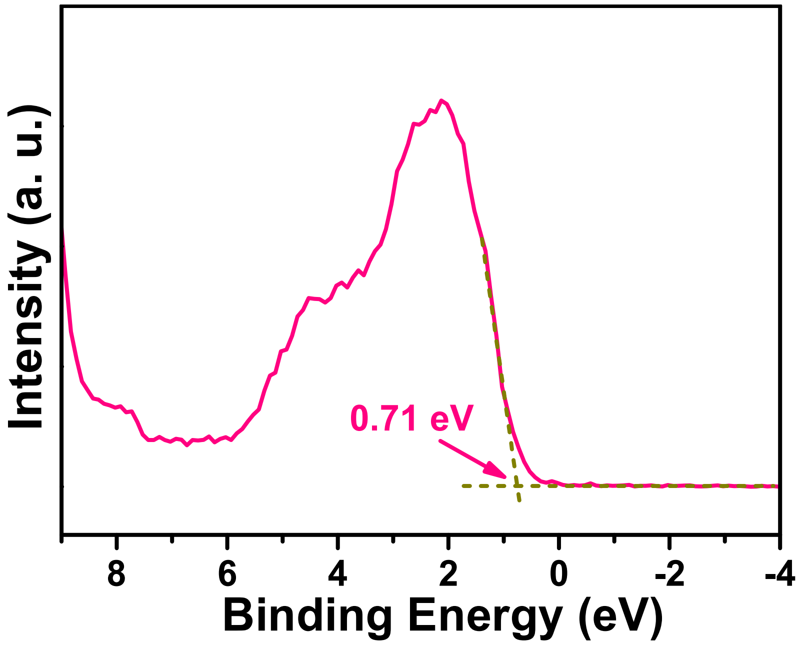

Supplementary Fig. 3 XPS valence band spectrum of the Cs_2SnI_6 powder. The valence-band edges can be obtained by depicting the tangential lines in the XPS valence band spectra of the curve around 0 eV. The reference work function is 4.75 eV.

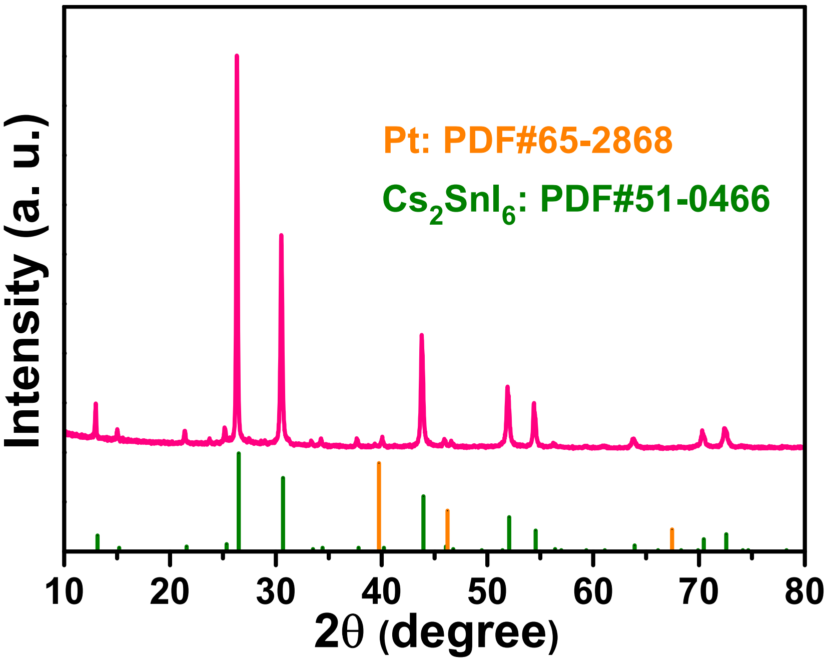

Supplementary Fig. 4 PXRD pattern of the PtSA/Cs_2SnI_6 powder.

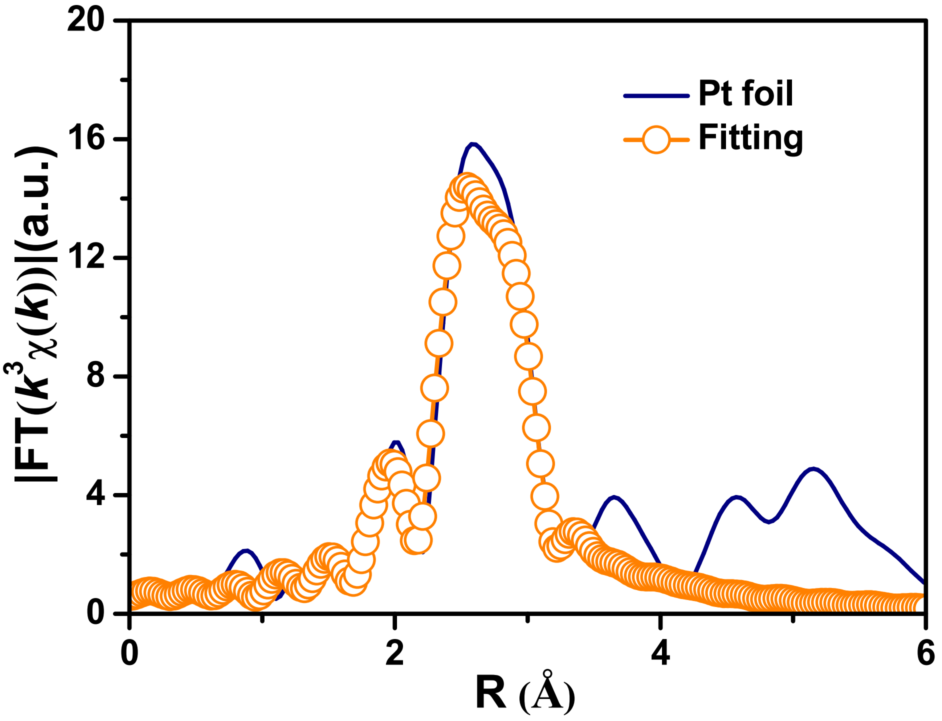

Supplementary Fig. 5 XANES R space fitting curves of Pt foil.

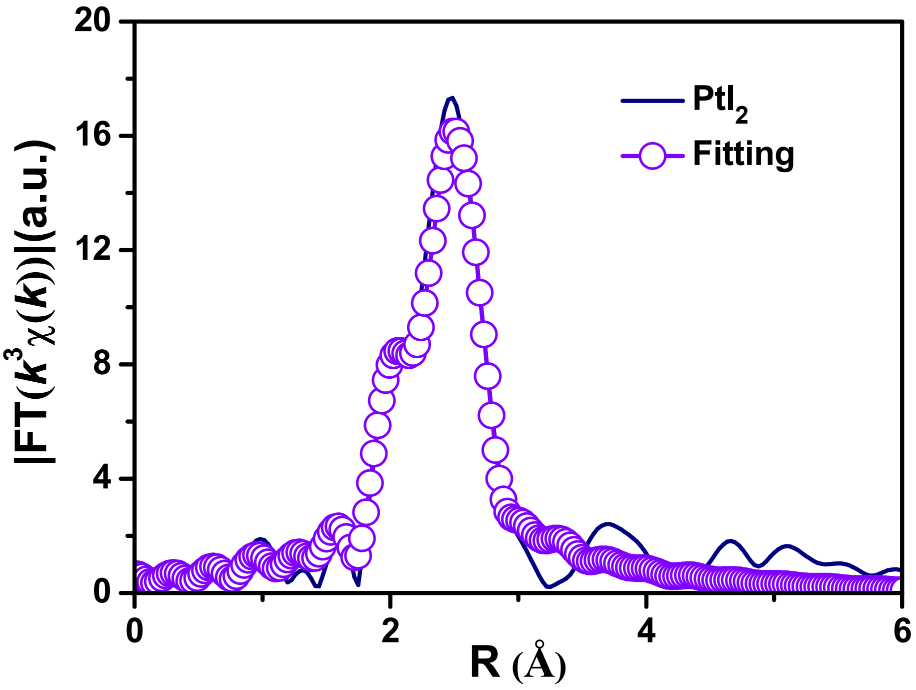

Supplementary Fig. 6 XANES R space fitting curves of PtI_2.

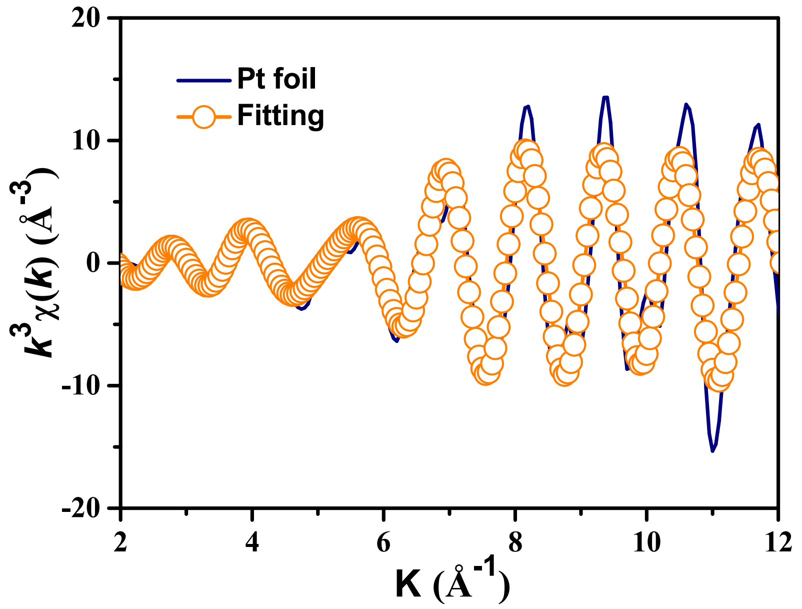

Supplementary Fig. 7 XANES K space fitting curves of Pt foil.

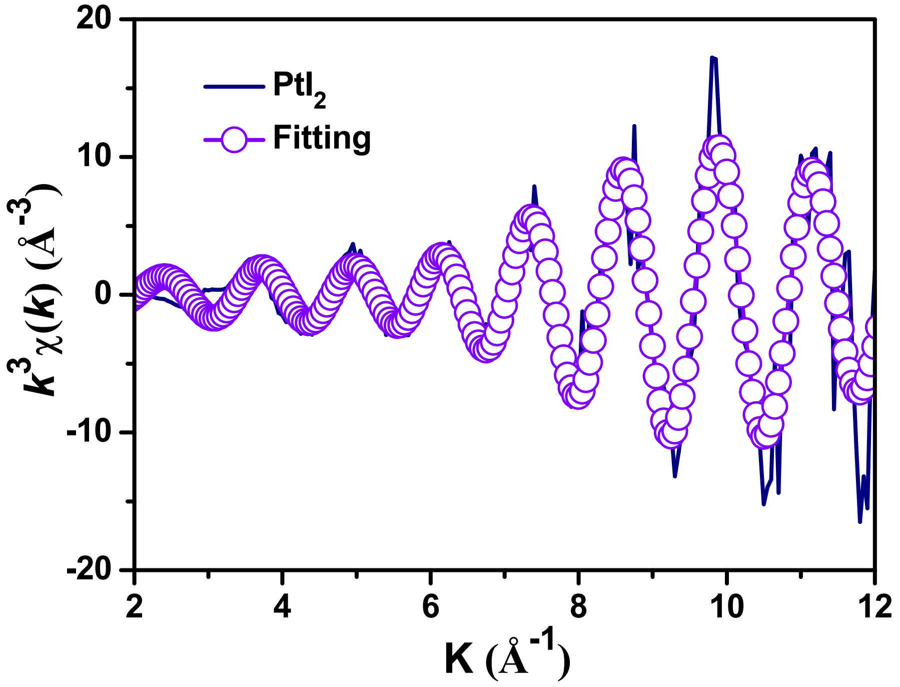

Supplementary Fig. 8 XANES K space fitting curves of PtI_2.

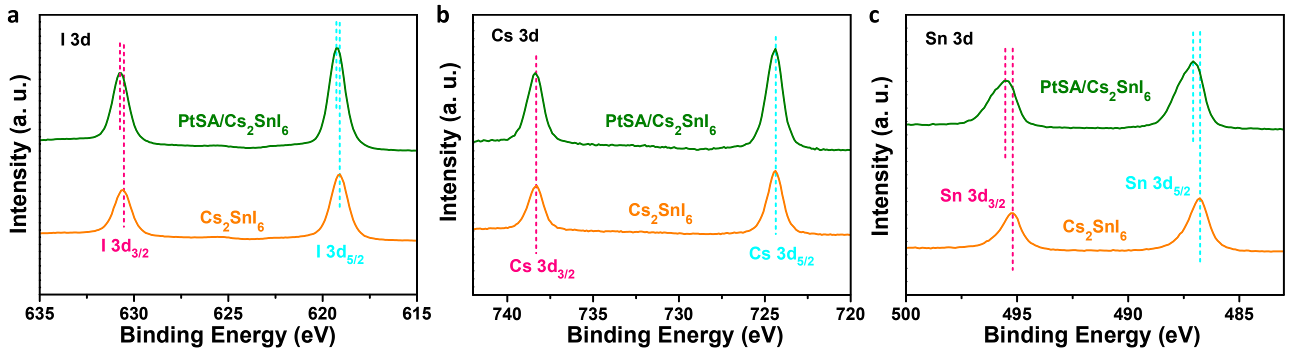

Supplementary Fig. 9 High-resolution XPS (a) I 3*d*, (b) Cs 3*d*, and (c) Sn 3*d* spectra of PtSA/Cs_2SnI_6 and Cs_2SnI_6.

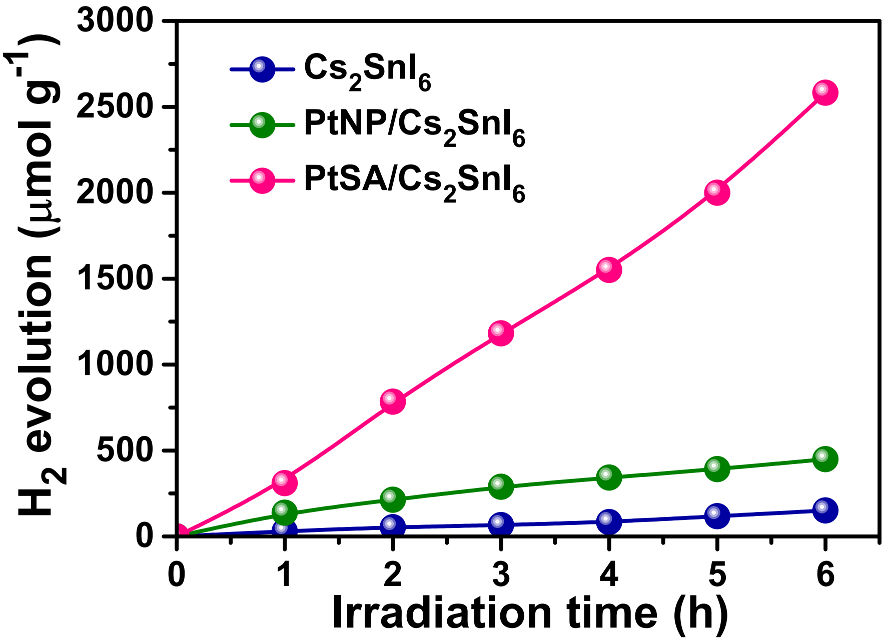

Supplementary Fig. 10 Time course of photocatalytic H_2 evolution performance of PtSA/Cs_2SnI_6 and reference catalysts.

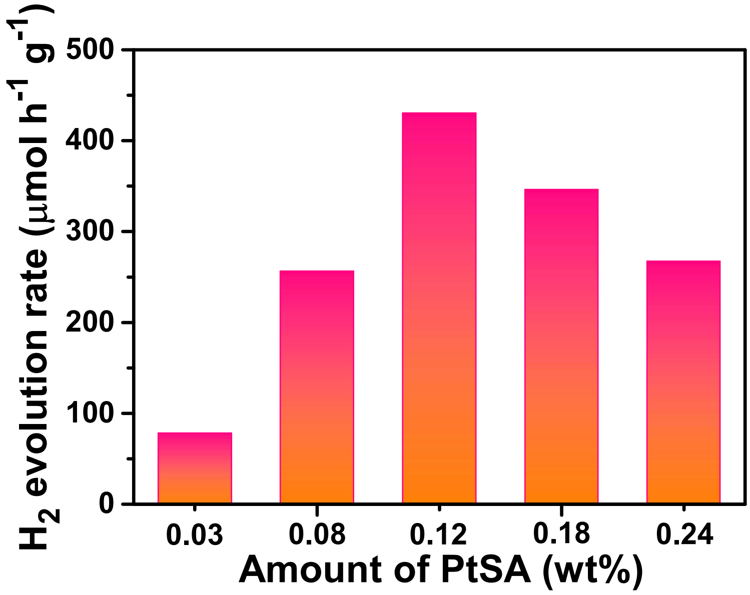

Supplementary Fig. 11 Photocatalytic H_2 evolution rate of PtSA/Cs_2SnI_6, which is dependent on the Pt loading amount.

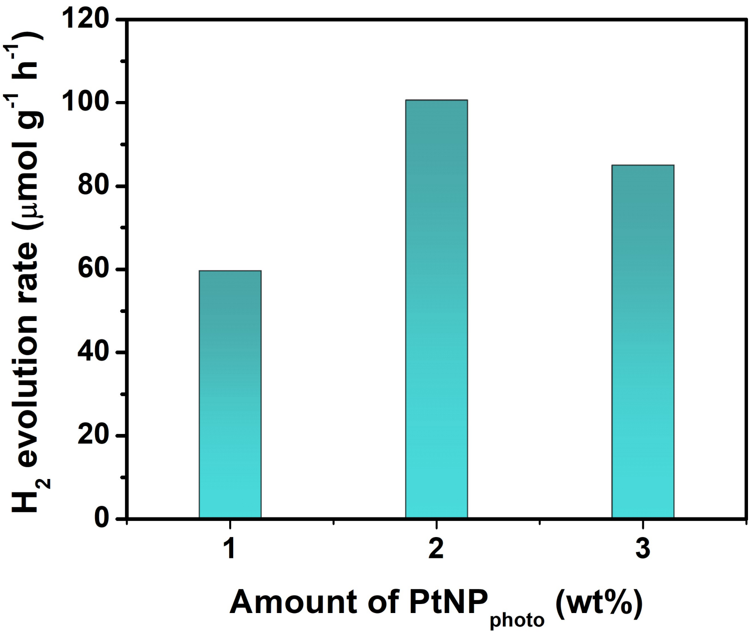

Supplementary Fig. 12 Photocatalytic H_2 evolution rate of $PtNP_{photo}/Cs_2SnI_6$, which is dependent on the loading amount of Pt.

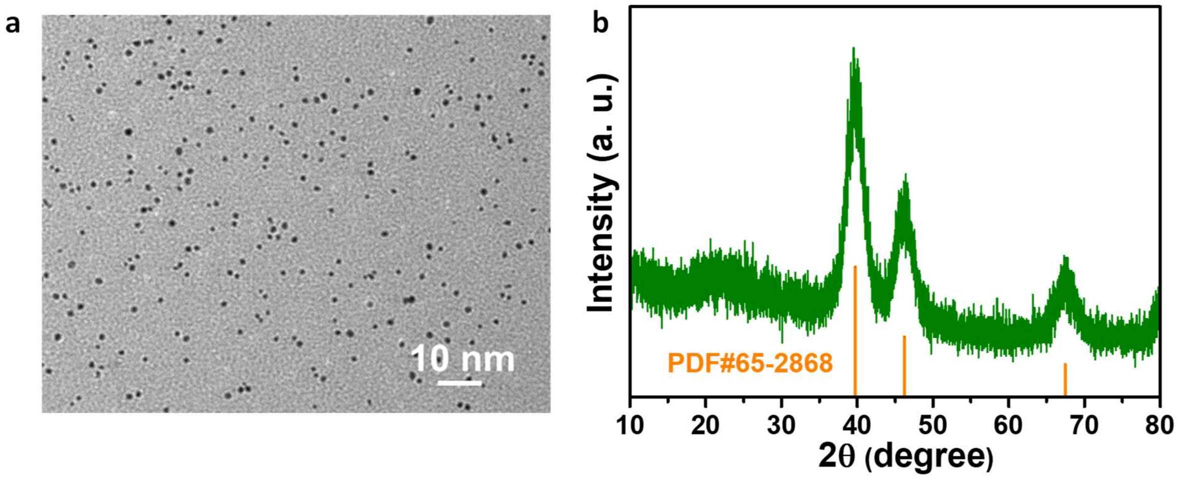

Supplementary Fig. 13 TEM image and PXRD pattern of the as-prepared Pt nanoparticles.

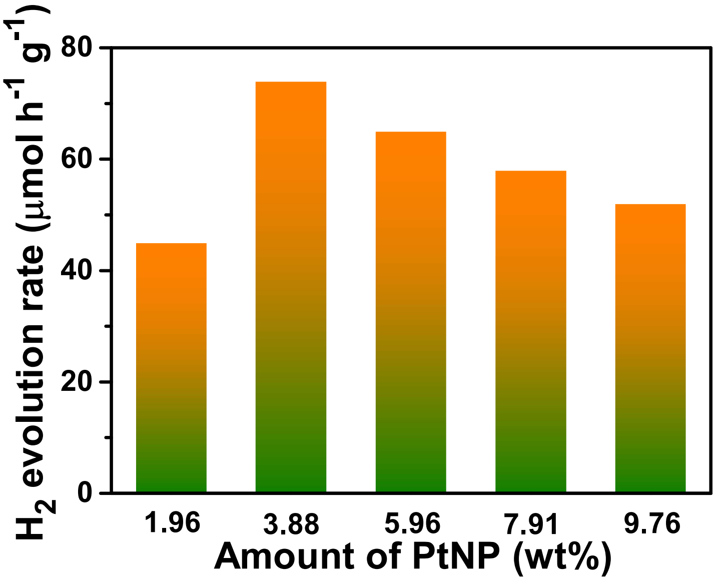

Supplementary Fig. 14 Photocatalytic H_2 evolution rate of PtNP/Cs_2SnI_6, which is dependent on the loading amount of Pt.

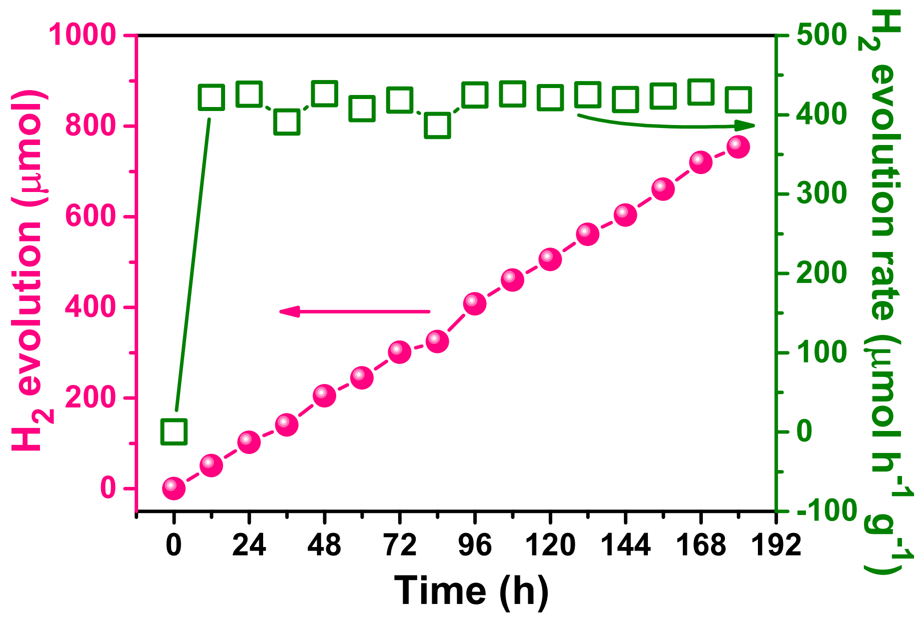

Supplementary Fig. 15 Long-term stability of PtSA/Cs_2SnI_6 over a 180 h period.

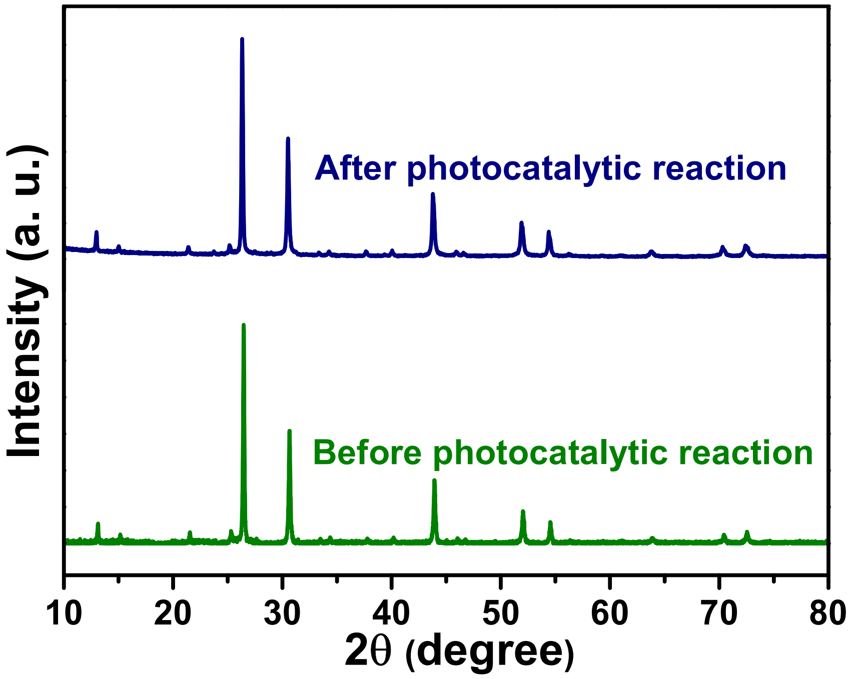

Supplementary Fig. 16 PXRD patterns of PtSA/Cs_2SnI_6 before and after stability test.

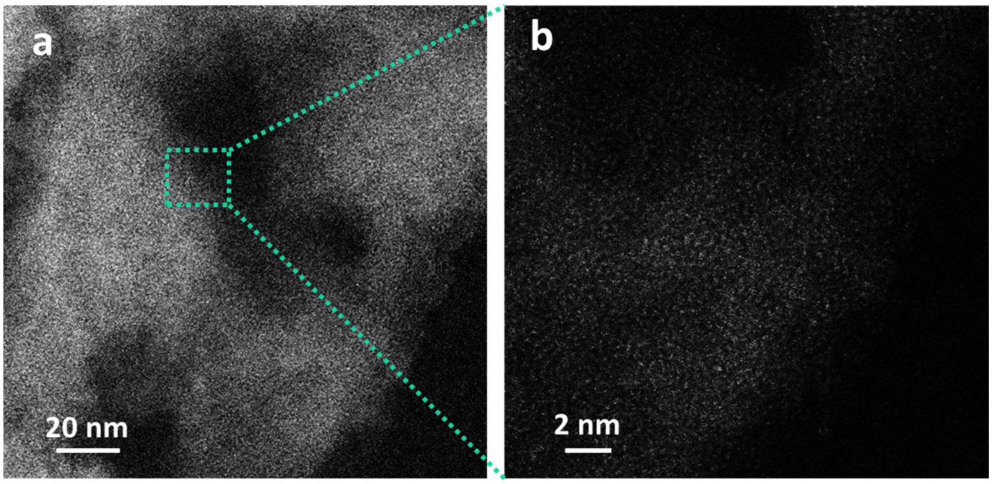

Supplementary Fig. 17 HAADF-STEM images of $PtSA/Cs_2SnI_6$ after stability test.

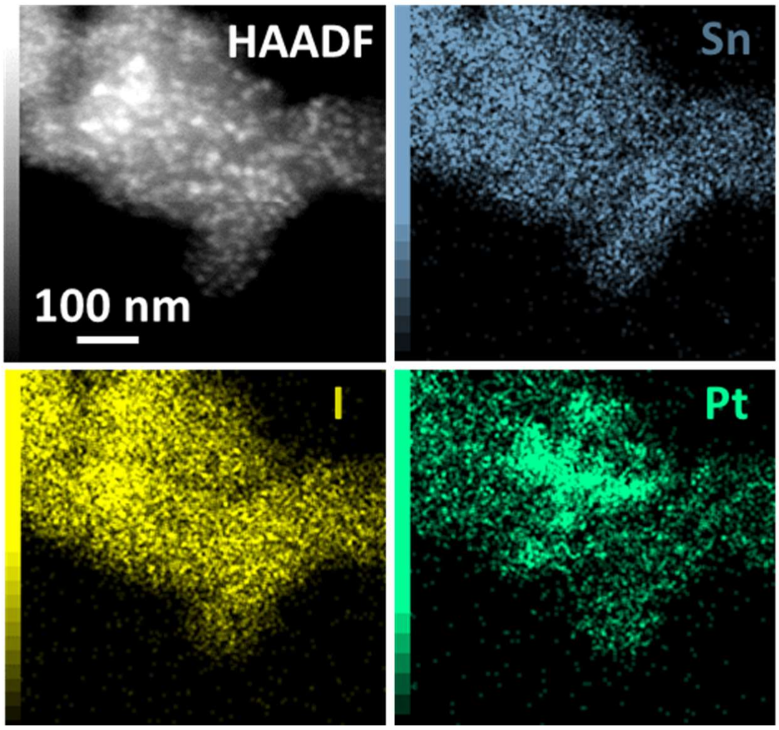

Supplementary Fig. 18 HAADF-STEM-EDS mapping images of PtSA/Cs_2SnI_6 after stability test.

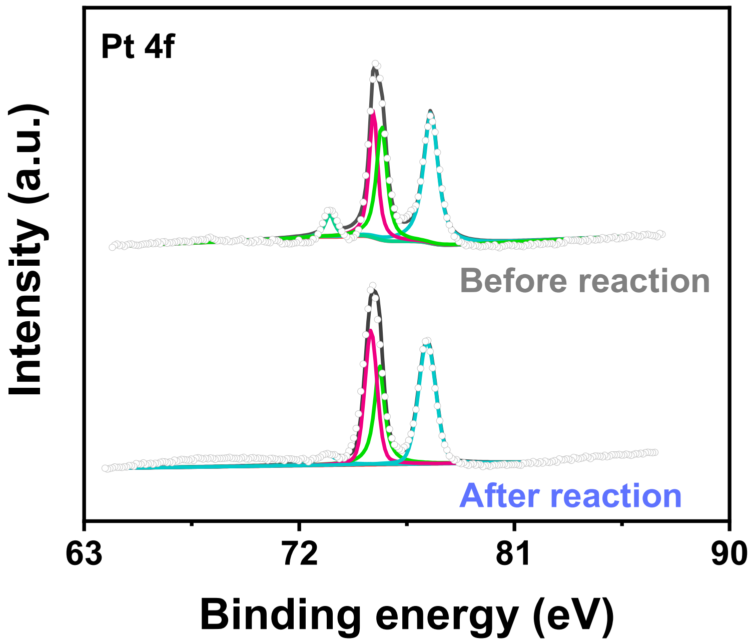

Supplementary Fig. 19 The XPS spectra of Pt 4*f* in PtSA/Cs_2SnI_6 before and after photocatalytic reaction.

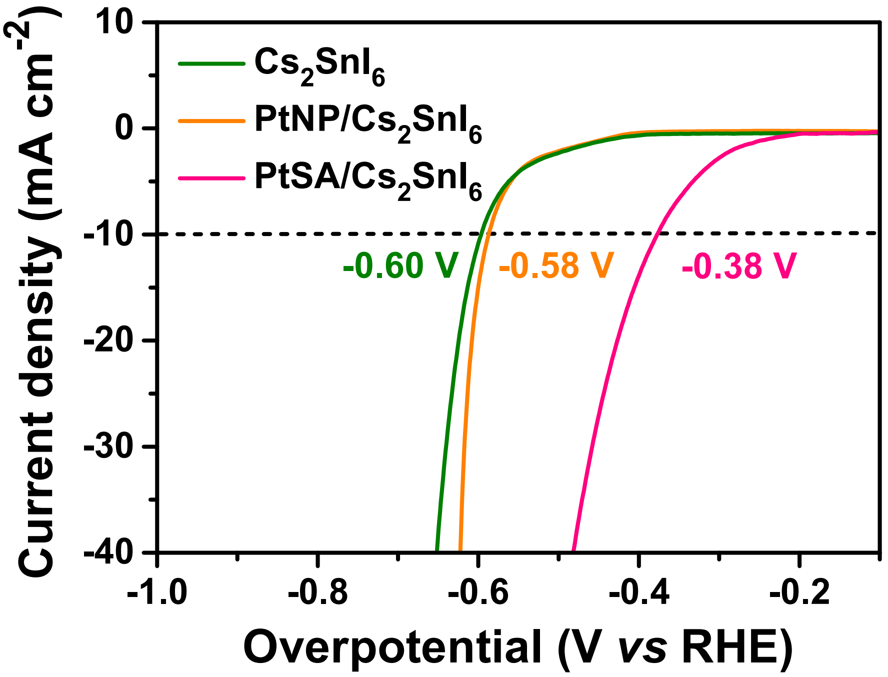

Supplementary Fig. 20 Linear-sweep voltammogram (LSV) curves of PtSA/Cs_2SnI_6 and references.

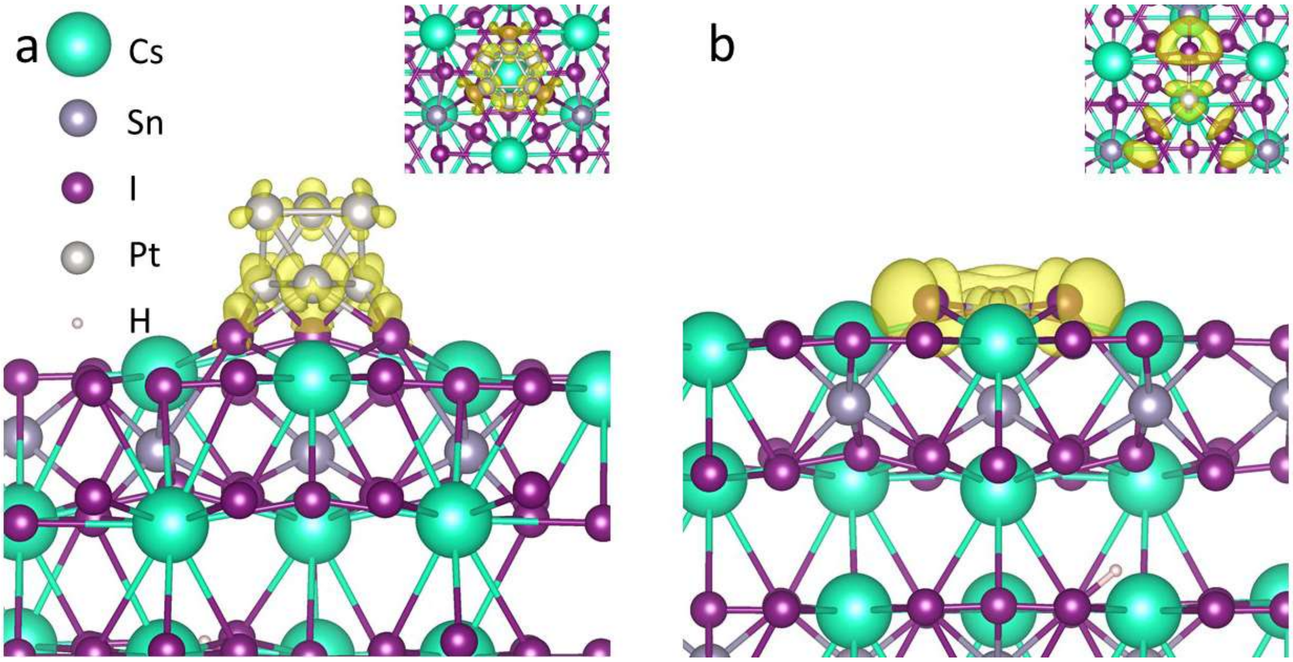

Supplementary Fig. 21 The charge density difference maps between PtSA/PtNP and Cs_2SnI_6: (a) PtNP/Cs_2SnI_6 and (b) PtSA/Cs_2SnI_6. The isosurface of charge density is 0.001 e Å^{-3}. The *insets* stand for the top view. The yellow region represents the additional electron distribution. An excess donor hydrogen atom was added into the models.

Supplementary Table 1. EXAFS fitting results of Pt in PtSA/Cs_2SnI_6 and references.

**Sample**	**Path**	**Coordination number**	**R (Å)**	**σ^2 (10^{-3} Å^2)**	**R-factor (10^{-3})**
PtSA/Cs_2SnI_6	Pt-I	3.33	2.66	1.31	6.10
Pt foil	Pt-Pt	9.86	2.76	4.64	1.87
PtI_2	Pt-I	4.55	2.66	4.09	5.53

Supplementary Table 2. The photocatalytic H_2 evolution activities comparison between 0.12 wt% PtSA/Cs_2SnI_6 and other reported Pt-loaded halide perovskite photocatalysts.

Photocatalysts	Light source	H_2 activity (μmol h^{-1} g^{-1})	TOF (h^{-1})	Reference
**0.12 wt% PtSA/Cs_2SnI_6**	**λ ≥ 420 nm 100 mW cm^{-2}**	**430**	**70.6**	**This work**
1.72 wt% PtNP/$MAPbI_3$	λ ≥ 475 nm 100 mW cm^{-2}	57	0.65	3
1.91 wt% PtNP/MAPbI3	λ ≥ 420 nm 120 mW cm^{-2}	40	0.41	4
1.22 wt% PtNP/$MAPbBr_{3-x}I_x$	λ ≥ 420 nm	2605	42.1	5
0.5 wt% PtNP/$MAPBr_3$	λ ≥ 420 nm 200 mW cm^{-2}	90	3.55	6
1.91 wt% PtNP/$(CH_3NH_3)_3Bi_2I_9$	λ ≥ 420 nm 100 mW cm^{-2}	169	1.75	7
0.75 wt% PtNP/$MAPBr_3$	λ ≥ 420 nm 150 mW cm^{-2}	20	0.53	8
6.0 wt% PtNP/$MAPbI_3$	λ ≥ 420 nm 100 mW cm^{-2}	534	1.75	9
1.2 wt% PtNP/$MAPbI_3$	λ ≥ 420 nm	192	3.15	10
2.5 wt% PtNP/$Cs_2AgBiBr_6$	λ ≥ 420 nm	1	0.02	11
3.0 wt% PtNP/$MAPbI_3$	380 nm ≤ λ ≤ 780 nm, 450 mW cm^{-2}	69	0.45	12
1.90 wt% Pt/$MAPbI_3$	λ ≥ 420 nm 100 mW cm^{-2}	575	5.96	13

Supplementary Table 3. Convergence tests with different slab thickness, vacuum thickness, PtNP sizes and supercell area.

**Slab thickness (CsI-Sn-CsI layer)**	**Slab vacuum thickness (Å)**	**Supercell area**	**Pt size**	***Van der Waals* functional correction[a]**	**ΔG (eV)**
3	16	2 × 2	Pt_1	No	-0.11
3	16	1 × 1	Pt_1	No	-0.50
3	16	3 × 3	Pt_1	No	-0.13
3	12	2 × 2	Pt_1	No	-0.22
3	20	2 × 2	Pt_1	No	-0.14
2	16	2 × 2	Pt_1	No	-0.24
4	16	2 × 2	Pt_1	No	-0.10
3	16	2 × 2	Pt_6	No	-0.91
3	16	2 × 2	Pt_{31}	No	-0.85
3	16	2 × 2	Pt_1	Yes	-0.15
3	16	2 × 2	Pt_6	Yes	-0.96

[a]DFT-D2 method was used in *Van der Waals* functional correction.

References

1. Han, X. *et al.* Lead-Free Double Perovskite Cs_2SnX_6: Facile Solution Synthesis and Excellent Stability. *Small* **15**, 1901650 (2019).
2. Chen, Y. *et al.* Engineering the Atomic Interface with Single Platinum Atoms for Enhanced Photocatalytic Hydrogen Production. *Angew. Chem. Int. Edit.* **59**, 1295-1301 (2020).
3. Park, S., Chang, W. J., Lee, C. W., Park, S., Ahn, H.-Y. & Nam, K. T. Photocatalytic hydrogen generation from hydriodic acid using methylammonium lead iodide in dynamic equilibrium with aqueous solution. *Nat. Energy* **2**, 16185 (2016).
4. Wu, Y. *et al.* Composite of $CH_3NH_3PbI_3$ with Reduced Graphene Oxide as a Highly Efficient and Stable Visible-Light Photocatalyst for Hydrogen Evolution in Aqueous HI Solution. *Adv. Mater.* **30**, 1704342 (2018).

5. Wu, Y. Q. *et al.* Enhancing the Photocatalytic Hydrogen Evolution Activity of Mixed-Halide Perovskite $CH_3NH_3PbB_{r3-x}I_x$ Achieved by Bandgap Funneling of Charge Carriers. *ACS Catal.* **8**, 10349-10357 (2018).
6. Wang, X. M. *et al.* Dynamic Interaction between Methylammonium Lead Iodide and TiO_2 Nanocrystals Leads to Enhanced Photocatalytic H_2 Evolution from HI Splitting. *ACS Energy Lett.* **3**, 1159-1164 (2018).
7. Guo, Y. M., Liu, G. N., Li, Z. X., Lou, Y. B., Chen, J. X. & Zhao, Y. X. Stable Lead-Free $(CH_3NH_3)_3Bi_2I_9$ Perovskite for Photocatalytic Hydrogen Generation. *ACS Sustain. Chem. Eng.* **7**, 15080-15085 (2019).
8. Wang, H. *et al.* Promoting Photocatalytic H_2 Evolution on Organic-Inorganic Hybrid Perovskite Nanocrystals by Simultaneous Dual-Charge Transportation Modulation. *ACS Energy Lett.* **4**, 40-47 (2019).
9. Zhao, Z. J., Wu, J. J., Zheng, Y. Z., Li, N., Li, X. T. & Tao, X. Ni_3C-Decorated $MAPbI_3$ as Visible-Light Photocatalyst for H_2 Evolution from HI Splitting. *ACS Catal.* **9**, 8144-8152 (2019).
10. Li, R. *et al.* Few-layer black phosphorus-on-$MAPbI_3$ for superb visible-light photocatalytic hydrogen evolution from HI splitting. *Appl. Catal. B-Environ.* **259**, 118075 (2019).
11. Wang, T., Yue, D., Li, X. & Zhao, Y. Lead-free double perovskite $Cs_2AgBiBr_6$/RGO composite for efficient visible light photocatalytic H_2 evolution. *Appl. Catal. B-Environ.* **268**, 118399 (2020).
12. Wang, F., Liu, X., Zhang, Z. & Min, S. A noble-metal-free MoS_2 nanosheet-coupled $MAPbI_3$ photocatalyst for efficient and stable visible-light-driven hydrogen evolution. *Chem. Commun.* **56**, 3281-3284 (2020).
13. Zhao, X. L. *et al.* Perovskite Microcrystals with Intercalated Monolayer MoS_2 Nanosheets as Advanced Photocatalyst for Solar-Powered Hydrogen Generation. *Matter* **3**, 935-949 (2020).
